# DCTPP1, an Oncogene Regulated by miR-378a-3p, Promotes Proliferation of Breast Cancer via DNA Repair Signaling Pathway

**DOI:** 10.3389/fonc.2021.641931

**Published:** 2021-05-25

**Authors:** Ming Niu, Ming Shan, Yang Liu, Yanni Song, Ji-guang Han, Shanshan Sun, Xiao-shuan Liang, Guo-qiang Zhang

**Affiliations:** ^1^Department of Breast Surgery, Harbin Medical University Cancer Hospital, Harbin, China; ^2^Research Institute of Chinese Medicine, Heilongjiang University of Chinese Medicine, Harbin, China

**Keywords:** breast cancer, oncogene, DCTPP1, miR-378a-3p, DNA repair

## Abstract

Breast cancer (BRCA) is one of the most deadly cancers worldwide, with poor survival rates that could be due to its high proliferation. Human all-alpha dCTP pyrophosphatase 1 (DCTPP1) is implicated in numerous diseases, including cancers. However, its role in BRCA is unclear. In this study, we used bioinformatic analyses of the ONCOMINE, UALCAN, and GEPIA databases to determine the expression pattern of DCTPP1 in BRCA. We found that elevated DCTPP1 levels correlate with poor BRCA prognosis. DCTPP1 silencing inhibited BRCA cell proliferation and induced apoptosis *in vitro*, as well as *in vivo*. Our data show that this tumorigenic effect depends on DNA repair signaling. Moreover, we found that DCTPP1 is directly modulated by miR-378a-3p, whose downregulation is linked to BRCA progression. Our results showed down-regulation of miR-378a-3p in BRCA. Upregulation of miR-378a-3p, on the other hand, can inhibit BRCA cell growth and proliferation. This study shows that reduced miR-378a-3p level enhances DCTPP1 expression in BRCA, which promotes proliferation by activating DNA repair signaling in BRCA.

## Introduction

Breast cancer (BRCA) is estimated to be responsible for 30% of all new cancer cases in women and is the second primary cause of cancer deaths in women (1–3). The poor prognosis and high death rates of BRCA are due to abnormal cellular proliferation (4). The standard treatments for BRCA patients include surgery, radiotherapy, and chemotherapy, or a combination of multiple methods. Although the overall survival rate of breast cancer patients has recently increased, tumor relapse, therapy resistance, or far-end metastasis still occur in several BRCA patients. The complex tumorigenic mechanisms of BRCA impede its treatment. Therefore, a better understanding of its underlying mechanisms and reliable markers is needed to improve BRCA outcomes.

Human all-alpha dCTP pyrophosphatase 1 (DCTPP1) localizes in the nucleus, cytosol, and mitochondria (5). DCTPP1 is highly expressed in embryonic tissue along with proliferative tissues with expanded nucleotide pools. This enzyme is upregulated also in various human cancers (6, 7). Song et al. showed that DCTPP1 promotes BRCA cell growth and stemness by modulating 5-methyl-dCTP metabolism and global hypomethylation (6). Zhang et al. suggested that nuclear DCTPP1 in cancer cells may suffice to maintain proper DNA replication, hence promoting survival and proliferation of BRCA cells (8). However, there is no evidence showing post-transcriptional regulation of DCTPP1 in BRCA progression.

MicroRNAs (miRNAs) are short oligonucleotides made of 22–25 nucleotides. miRNAs can act as a tumor suppressor to inhibit cancer progression by modulating proliferation, apoptosis, invasion/metastasis, as well as angiogenesis (9–11). miRNAs dock to the 3′-UTR of target genes to modulate the expression of at least 30% of all protein-coding genes (12). In BRCA, they have oncogenic and anti-cancer roles depending on their target messenger RNA (mRNA) (13, 14). Numerous studies have shown that miRNAs influence BRCA cancer proliferation, apoptosis, and resistance to chemotherapy. For instance, miR-21 and miR-210 upregulation in BRCA correlates with poor prognosis (15). The miR-200 family includes miR-200a, miR-141, miR-200b, miR-429, and miR-200c. Expression of these miRNAs is lost in invasive BRCA cell lines (16). However, there is less evidence that miR-378-3p is involved in BRCA. miR-378a-3p has anti-tumor effects in glioblastoma multiforme through targeting tetraspanin 17 (17). miR-378a-3p also sensitizes ovarian cancer cells to cisplatin via MAPK1/GRB2 signaling (18). In BRCA cells, miR-378a-3p serves as a biomarker in age-related BRCA and BRCA evolution during adjuvant chemotherapy (19). However, the precise mechanisms underlying the function of miR-378a-3p in BRCA are unclear.

DNA repair is critical for genomic integrity and is activated by DNA damage, making it critical for cell survival (20). Zhang et al. suggested that nuclear DCTPP1 in cancer cells might suffice to maintain proper DNA replication, promoting survival, and proliferation of BRCA cells (21). There is no evidence of DCTPP1 involvement in DNA repair in BRCA cells.

Here, we found that DCTPP1 overexpression in BRCA cells and tissues correlates with a poor prognosis. The present study demonstrated that DCTPP1 enhanced BRCA cell proliferation and that the miR-378a-3p direct target is downregulated in BRCA. On the other hand, it repressed proliferation when upregulated. DNA repair signaling cascade is a primary signaling axis of miR-378-3P/DCTPP1 in BRCA with a poor prognosis. Our findings highlight miR-378-3P/DCTPP1 signaling as a potential therapeutic target against BRCA.

## Methods

### Cell Lines and Cell Culture

Human BRCA cell lines MDA-MB-231, MDA-MB-468, MCF-7, and BT-549 as well as normal breast epithelial cells MCF-10A were purchased (22). BRCA cells were grown in complete high glucose DMEM (Wisent, USA), enriched with 10% FBS, 100 μg/ml pen/strep (Hyclone, USA). MCF10A were cultured in mammary epithelial cell basal medium (MEBM, Lonza, USA) supplemented with 100 ng/ml cholera toxin. All cells were grown at 37°C, 5% CO_2_ in a humidified incubator.

### Lentiviral Transfection and Small Interfering RNA

DCTPP1 knockdown (shDCTPP1) or DCTPP1 overexpression (DCTPP1), and a scrambled sequence (SCR) or a negative control (NC) sequence, respectively, were used according to the manufacturer's instructions. Plasmid sequences were validated via sequencing (GenePharma, China). We cultured the cells were in 6-well plates at 30% confluence, followed by inoculation with the retroviruses. Polybrene (5 μg/ml) was used to enhance infection efficiency. Stably transfected cells (puromycin-resistant) were selected by treating with puromycin (2 μg/ml) for 2 weeks. DCTPP1-overexpressing MCF7 and MDA-MB-468 cells, and control (NC) cells were inoculated into 6-well plates and cultured overnight. Next, siRNA (GenePharma, China) and non-targeting control siRNA were transfected using lipofectamine® 3000 (Invitrogen, USA) using manufacturer instructions. The sequences of the siRNAs were: Sense 5′-gatccgcccttcaagaggagcttattcaagagataagctcctcttgaagggcttttttacgcgt g-3′ and antisense 5′-aattcacgcgtaaaaaagcccttcagaggagcttatctcttga agctcctcttgaagggcg-3′. miRNA sequences were as follows: miR-378a-3p mimic: cuggacuuggagucagaagg, mimic-NC: agugcauguuaugccuacg, miR-378a-3p inhibitor: aguucagguucugacuccu, inhibitor-NC: ugguccguguaggccuacua.

### RT-qPCR Analysis

Isolation of total RNA was carried out with the Trizol reagent (TaKaRa, USA). cDNA was generated from 1 μg of RNA via reverse-transcription with the Primescript RT Reagent (TaKaRa, USA). The FastStart Universal SYBR Green Master (Roche, USA) was employed to perform RT-qPCR on a real-time PCR instrument (Applied Biosystems, USA). GAPDH and U6 were used as reference genes. The primers are indicated in Supplementary Table 1.

### Western Blot Assessment

Cells were lysed using RIPA buffer (Thermo Fisher, USA) enriched with 0.1% protease inhibitor, 1% phosphatase inhibitor, as well as 1% PMSF. Fractionation of the proteins was done on SDS-PAGE gel and then the proteins transfer-embedded onto NC membranes (Millipore, USA). Afterward, membranes were blocked with 5% skimmed milk in PBS for 2 h, followed by overnight incubation with anti-β-actin (Cell Signaling Technology, USA) and anti-DCTPP1 (Abgent Inc., USA) at 4°C. They were then washed thrice, 10 min each with PBST, and incubated for 1 h with indicated secondary antibodies.

### Cell Counting Kit (CCK-8) Assay

Cell proliferation was examined using a CCK-8 kit (Dojindo, Japan) as per the manufacturer's protocol. 2 × 10^3^ cells/well, in 200 μl of cell culture media, were cultured onto 96-well plates and cultured at 37°C for 4 h. The cell culture medium was then replaced with media enriched with 10% CCK8, then incubation of the cells was performed at 37°C for 2 h. Thereafter, a microplate reader was employed to determine the absorbance at 450 nm.

### Ki67 Assay

Cells were seeded in 6-well plates before transfection with DCTPP1-siRNA, mimic of miR-378a-3p, or their NC control for 48 h. After that, fixation with 75% absolute ethanol was performed for 60 s, followed by rinsing twice with PBS, then staining by Giesma (Sigma, USA) for 15 min, and dried at room temperature. The colonies with ≥50 cells/well were then counted.

### TUNEL Assay

Breast cancer cells (MCF-7 and MDAMB-468) were inoculated in 6-well plates before transfection with DCTPP1-siNRA or control group for 48 h. TUNEL assay kit (Roche, Germany) was used to test MCF-7 and MDA-MB-468 cell apoptosis. After that, cells fixation with 4% PFA (paraformaldehyde) for 15 min. Then, MCF7 and MDA-MB-468 were blocked in 0.1% Triton X-100 for 1 h. Cells were then treated with a TUNEL reaction mixture at room temperature for 1 h. Cell nuclei were stained by DAPI for 5 min.

### Dual-Luciferase Assay

Where specified, cells were inoculated and grown in triplicates for 24 h, followed by co-transfection with DCTPP1-3′-UTR clones or mutant clones with pRL-TK Renilla plasmid and miR-378a-3p mimic. After the elapse of 48 h, the luciferase enzyme activity of transfected cells was assessed using a dual luciferase assay kit (Promega, USA) following the manufacturer's instructions.

### *In vivo* Tumor Xenograft Model

All animal experiments adhered to guidelines by the Institutional Animal Care and Use Committee of the Harbin Medical University. We randomly split 28, 4-week old female BALB/c nude mice, weighing 18–22 g into four groups. Stable DCTPP1-siRNA, miR-378a-3p mimic, NC MCF-7 cells, or control cells (1 × 10^6^ cells in 100 μl of PBS) were subcutaneously administered into mammary fat pads of the mice, and tumor volume measured weekly using calipers. Tumor volume was given by the formula: (tumor length × width × height)/2. After 6 weeks, we sacrificed the mice and measured the final tumor weight.

### Clonogenic Survival Assay

We transfected the 8 × 10^2^ MCF-7, and MDA-MB-468 cells with DCTPP1 siRNA, control siRNA, miR-378a-3p mimic, or mimic of miR-NC were planted into 6 cm dishes and cultured for 10 d. Staining of colonies by 0.1% crystal violet in 20% methanol was done for 15 min. Five-hundred cells as the standard for a Clonogenic. They were then imaged, and visible colonies determined.

### γH2A Immunofluorescence (IF) Staining

MCF-7 and MDA-MB-468 cells were seeded onto glass coverslips and grown for 24 h to 50–60% density. They were then treated with DCTPP1 siRNA, or Control-si, for 48 h and fixed. They were then stained using anti-γH2A antibody (CST, USA) for 1 h and then incubation with secondary antibody performed for 20 min at RT. They were then counterstained with DAPI and mounted on prolong® diamond antifade (Applied biosystems, USA).

### Database Analysis

To determine DCTPP1 transcription levels in breast cancer, we analyzed gene expression cohorts in the ONCOMINE (https://www.oncomine.org), UALCAN (http://ualcan.path.uab.edu/analysis.html), Gene Expression Profiling Interactive Analysis (GEPIA) (http://gepia.cancer-pku.cn/index.html). Metascape (http://metascape.org) databases to explore the interaction and function of DCTPP1 co-expressed genes. Enrichment of GO terms including biological process, cellular component, and molecular function, and KEGG pathways was carried on using the Metascape online tools. Functional terms with *p*-value ≤ 0.01 and minimum count ≥3 were considered statistically significant. The most significant term within each cluster was chosen as representative of this cluster. Then, the association between the significant terms was established as a network, in which terms with similarities >0.3 were connected. Protein–protein interaction enrichment assessment was done on BioGrid, InWeb_IM, as well as OmniPath. The Molecular Complex Detection (MCODE) algorithm was used to determine the densely connected network components.

### Statistical Analysis

All experiments were done three times unless otherwise indicated. Data were analyzed using the SPSS 20.0 (IBM). For continuous variables, the Students *t*-test was employed to establish statistically remarkable differences between groups. *P* < 0.05 signified statistical significance.

## Results

### *DCTPP1* Expression in BRCA

**Figure 1:**
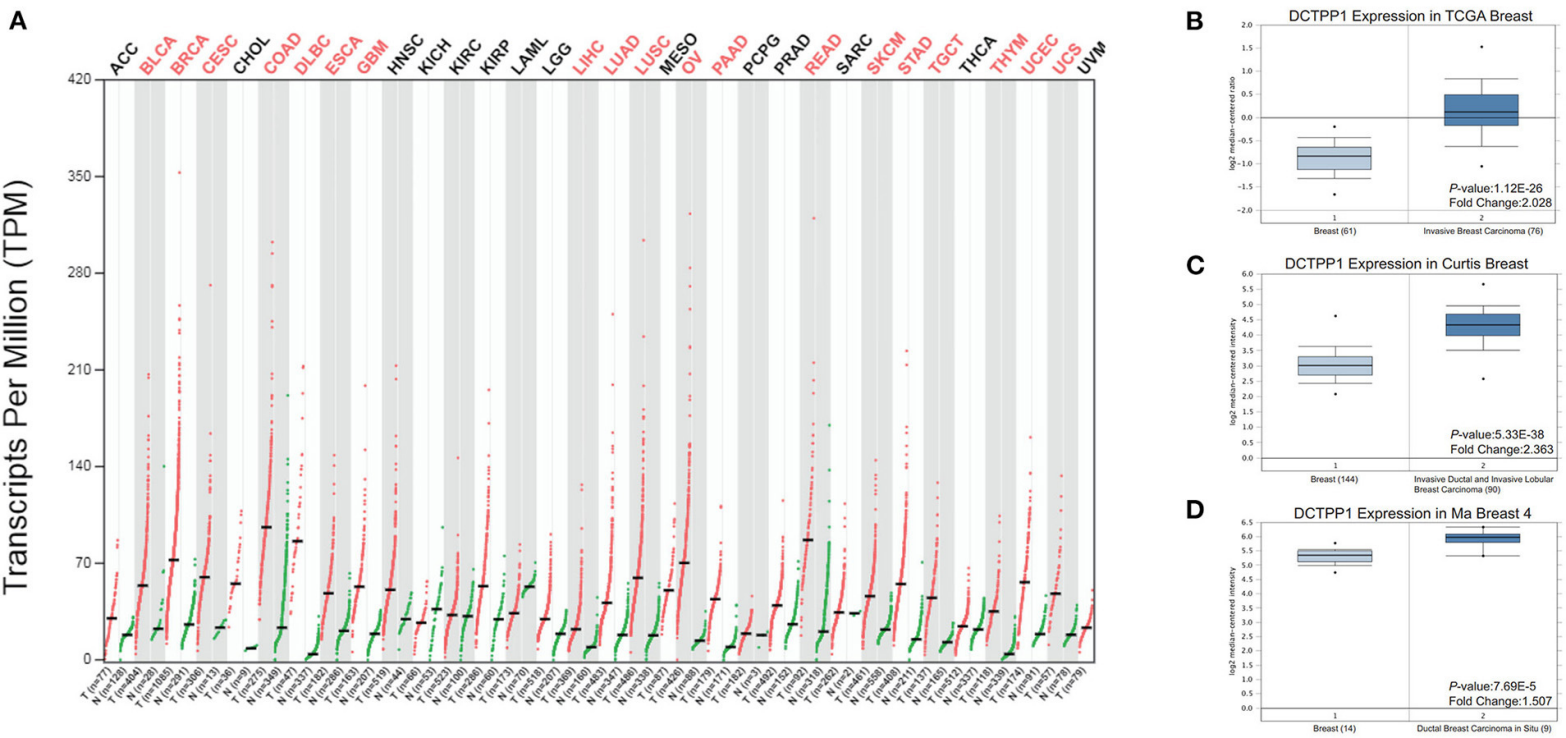
*DCTPP1* expression in BRCA. **(A)** *DCTPP1* expression in pan-cancer. **(B)** *DCTPP1* expression in TCGA breast cancer. **(C)** *DCTPP1* expression in Curtis breast cancer. **(D)** *DCTPP1* expression in Ma breast 4 cancer.

**Figure 2:**
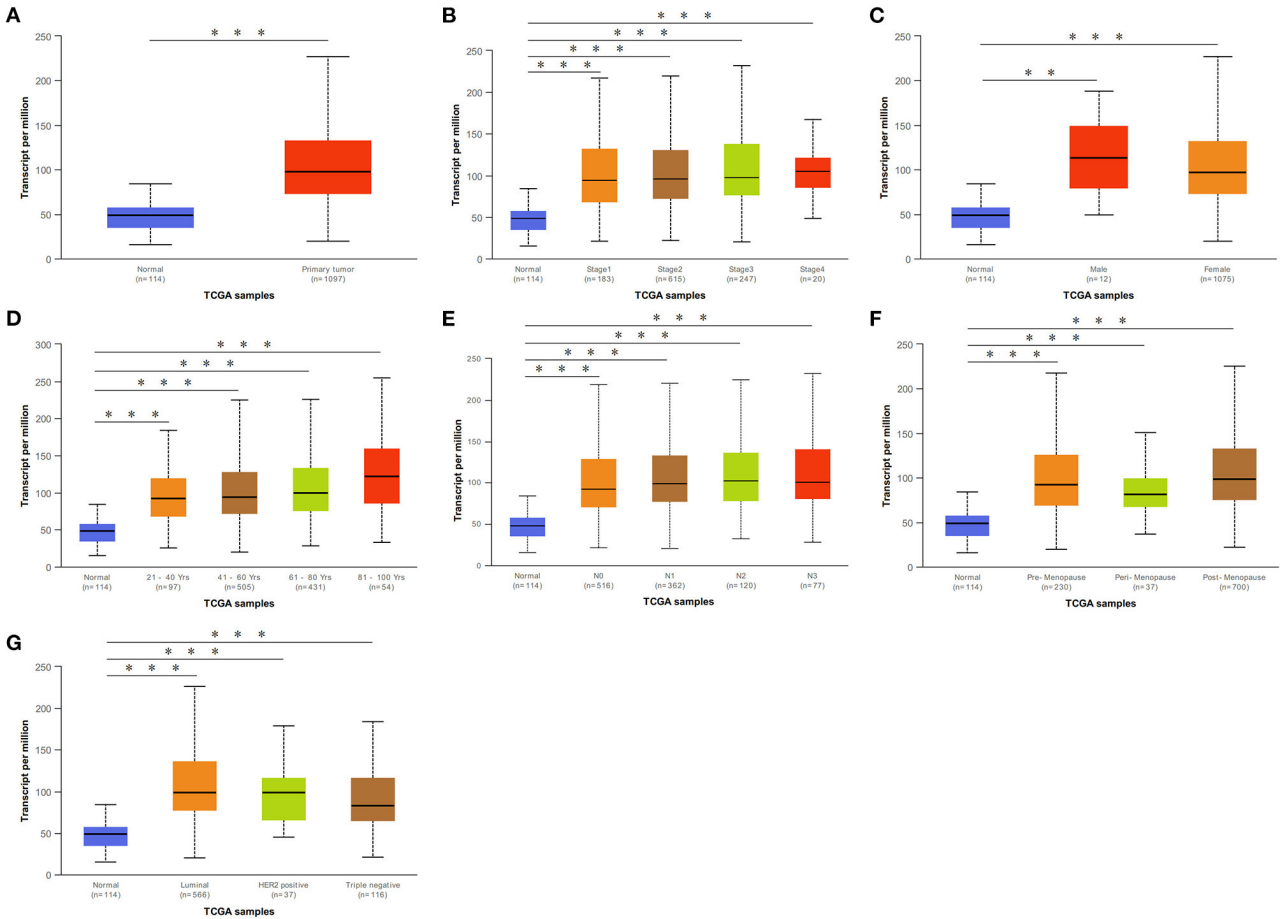
*DCTPP1* in BRCA patients was higher than that of healthy people. **(A)** *DCTPP1* expression in normal breast tissues and BRCA. **(B)** *DCTPP1* expression in I–IV stage of BRCA. **(C)** *DCTPP1* expression in male and female of BRCA. **(D)** *DCTPP1* in different age group of BRCA. **(E)** *DCTPP1* expression in different lymph node metastasis of BRCA. **(F)** *DCTPP1* expression in different menopause of BRCA. **(G)** *DCTPP1* expression in different molecular subtypes of BRCA. **p ≤ 0.01, ***p ≤ 0.001.

We initially evaluated DCTPP1 transcriptional expression in multiple BRCA datasets on TCGA and Gene Expression Omnibus (GEO). Figure 1A shows the DCTPP1 expression profile in 33 cancers (GEPIA). Analysis of DCTPP1 expression in three Oncomine datasets relative to normal tissues revealed DCTPP1 overexpression in BRCA tissue relative to normal breast tissue (Figures 1B–D, *p* ≤ 0.01). Fold differences were all >1.5. Analysis of DCTPP1 expression in BRCA tumors vs. normal tissues using UALCAN revealed that regardless of age, gender, disease stage, nodal metastasis, major subclasses, or menopause status, DCTPP1 transcription levels were remarkably elevated in BRCA patients than in healthy controls (Figure 2). Thus, DCTPP1 has diagnostic potential in BRCA.

### *DCTPP1* Has Prognostic Potential in BRCA

**Figure 3:**
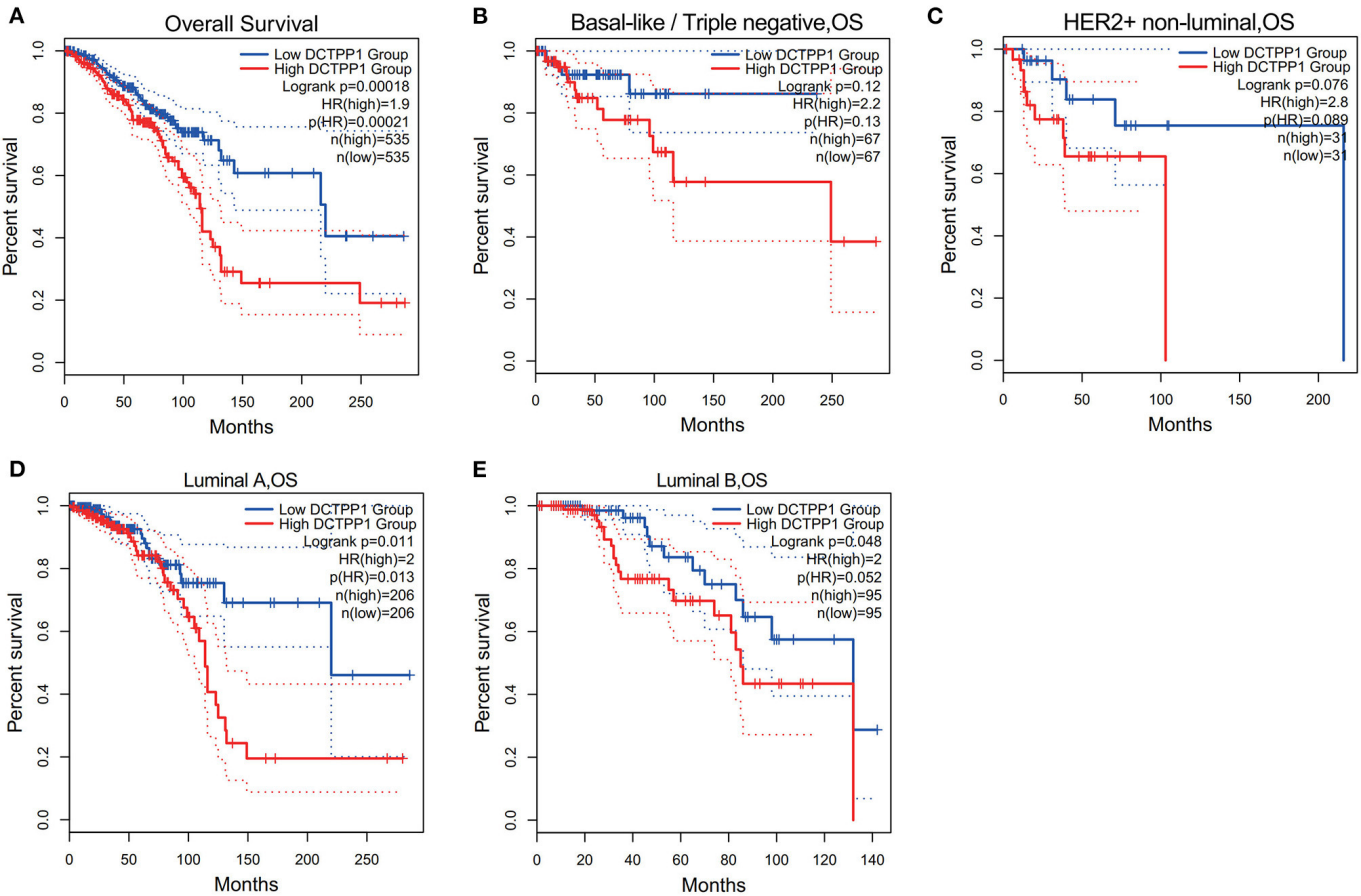
Prognostic potential of *DCTPP1* in BRCA. **(A)** Correlation of *DCTPP1* expression and OS of BRCA. **(B)** Correlation of *DCTPP1* expression and OS of Basal-like/Triple negative BRCA. **(C)** Correlation of *DCTPP1* expression and OS of HER2+/non-luminal BRCA. **(D)** Correlation of *DCTPP1* expression and OS of Luminal A BRCA. **(E)** Correlation of DCTPP1 expression and OS of Luminal B BRCA.

We then investigated if *DCTPP1* expression correlates with BRCA prognosis. The impact of *DCTPP1* expression on survival was evaluated using GEPIA. Notably, *DCTPP1* expression significantly impacts BRCA prognosis [OS HR = 1.9, Logrank *p* = 0.00018, *p*(HR) = 0.00021; Figure 3A]. Analysis of the effects of DCTPP1 expression on the prognosis of BRCA subtypes demonstrated that high DCTPP1 expression levels were significantly linked to the prognosis of Luminal A and B subtypes [Luminal A: OS HR = 2, Logrank *p* = 0.011, *p*(HR) = 0.013, Luminal B OS HR = 2, Logrank *p* = 0.048, *p*(HR) = 0.052; Figures 3D,E]. However, DCTPP1 levels did not correlate with prognosis of basal-like and HER2+, non-luminal subtypes [basal-like/triple-negative OS HR = 2.2, Logrank *p* = 0.12, *p*(HR) = 0.13, HER2+, non-luminal OS HR = 2.8, Logrank *p* = 0.076, *p*(HR) = 0.089; Figures 3B,C].

### Silencing of DCTPP1 Suppressed MCF-7 and MDA-MB-468 Proliferation and Induced Apoptosis

**Figure 4:**
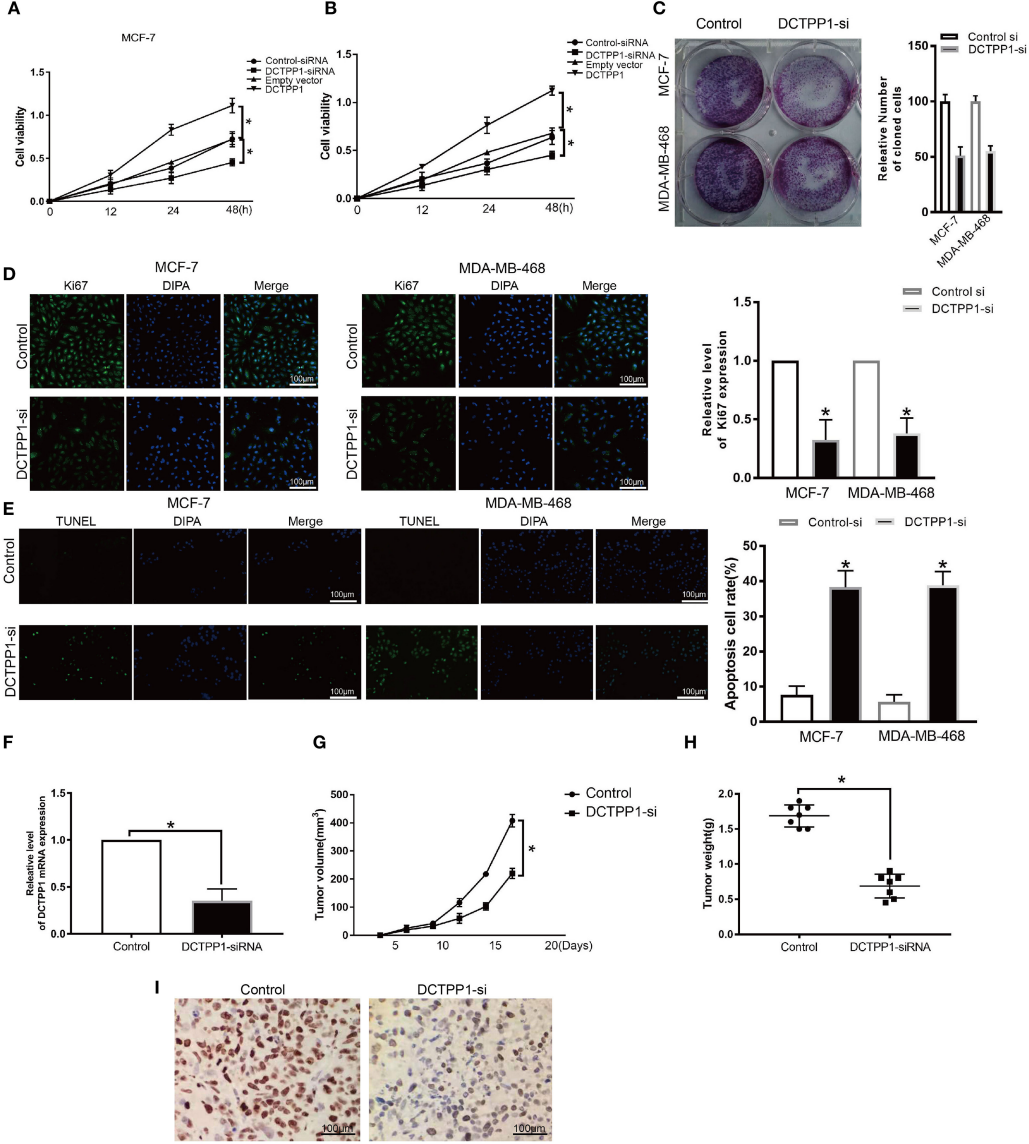
Silencing of DCTPP1 suppressed MCF-7 and MDA-MB-468 proliferation and induce apoptosis. **(A,B)** CCK-8 analysis of BRCA cell growth after DCTPP1 knockdown or overexpression. **(C)** Colony formation assays were used to test BRCA cell proliferation upon DCTPP1 silencing. **(D)** Ki67 analysis of BRCA cell proliferation upon DCTPP1 silencing. **(E)** TUNEL staining of BRCA cell apoptosis treat with DCTPP1 silencing. **(F)** DCTPP1 expression in DCTPP1-deficient tumors. **(G)** Tumor weight. **(H)** Tumor volume. **(I)** Ki67 levels in tumor tissues. *p ≤ 0.05 vs. control (*t*-test).

To explore the role of DCTPP1 in BRCA cancer, we first test the expression of DCTPP1 in different BC cell lines include the TNBC cell line. We found that DCTPP1 is up-regulated in MCF-7 and MDA-MB-468 (Supplementary Figure 1). To assess DCTPP1 effects on proliferation, it was overexpressed or silenced in MCF-7 and MDA-MB-468. Western blot and RT-qPCR analysis verified transfection efficiency (Supplementary Figures 2A–D). CCK-8 analysis of DCTPP1 effects on cell growth revealed that DCTPP1 silencing suppressed MCF-7 and MDA-MB-468 proliferation (Figures 4A,B). The clonogenic analysis revealed that DCTPP1 knockdown suppressed BRCA tumorigenic potential (Figure 4C). Additionally, KI67 staining was used to assess the effect of DCTPP1 on BRCA proliferation and revealed that DCTPP1 silencing suppresses BRCA cell proliferation (Figure 4D). TUNEL assay was used to test the cell apoptosis after treatment with DCTPP1 inhibition. Our data showed that silencing DCTPP1 can induce MCF-7 and MDA-MB-468 cell apoptosis (Figure 4E). Together, these data indicate that DCTPP1 silencing suppresses BRCA cell proliferation *in vitro*.

### *DCTPP1* Repressed Tumorigenesis *in vivo*

To explore the impact of DCTPP1 in BRCA *in vivo*, DCTPP1-deficient MCF-7 and controls were transplanted into the mammary fat pads of mice. Our analysis revealed low DCTPP1 levels in DCTPP1-siRNA tumor tissue relative to control tissue (Figure 4F). Moreover, DCTPP1-siRNA tumor volume growth was slower relative to control tumors (Figure 4G). At 6 weeks, DCTPP1-siRNA tumor weights were significantly lighter than control tumors (Figure 4H). Moreover, DCTPP1-siRNA tumors expressed lower Ki67 levels relative to controls (Figure 4I). Together, these data show that DCTPP1-knockdown suppresses tumorigenesis *in vivo*.

### *DCTPP1* Silencing Activates DNA Repair Mediated Signaling Pathway

**Figure 5:**
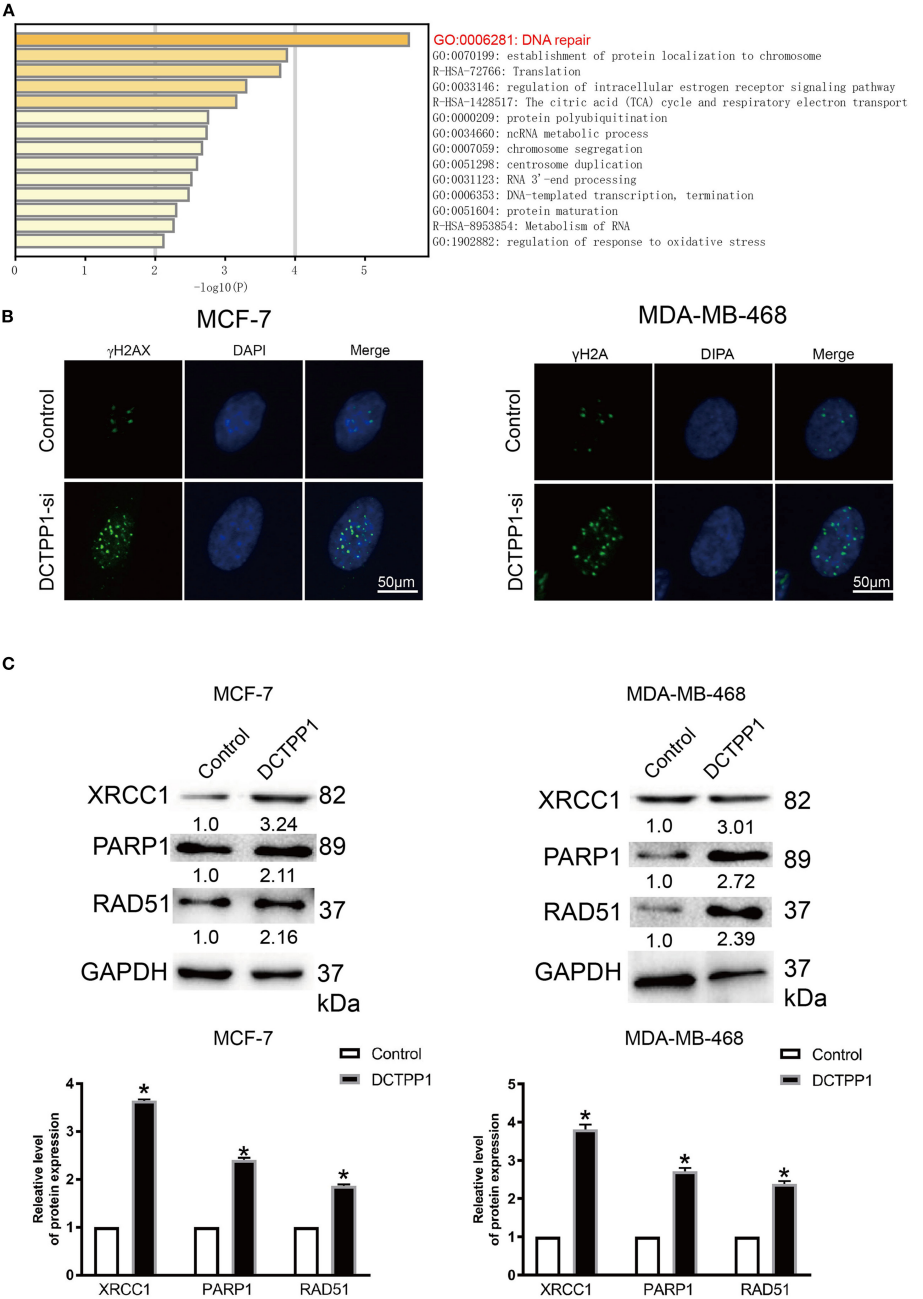
*DCTPP1* regulates DNA repair signaling. **(A)** KEGG pathway analysis. **(B)** γ-H2A analysis of DNA damage upon DCTPP1-silencing in BRCA cells. **(C)** Western blot analysis revealed elevated XRCC1, PARP1, and RAD51 levels upon DCTPP1-over-expression. The assay was repeated in BRCA cells. *T*-test, **p ≤ 0.01, *p ≤ 0.05 in contrast with the control group.

To investigate the biological role of *DCTPP1* in BRCA, the top 100 genes co-expressed with *DCTPP1* TCGA BRCA datasets were obtained. Metascape analysis was used to identify the pathways and processes enriched in all co-expression genes, including GO biological processes as well as reactome gene sets. Functional terms with *p* ≤ 0.01 and a minimum count of three were selected. Co-expressed genes, were mainly enriched in GO BP terms like DNA repair, protein localization to chromosome, and regulation of intracellular estrogen receptor signaling (Figure 5A and Table 1).

**Table 1:** Functional terms enriched by DCTPP1 co-expressing genes.

**GO**	**Category**	**Description**	**–Log10(*P*)**
GO:0006281	GO biological processes	DNA repair	5.63
GO:0070199	GO biological processes	Establishment of protein localization to chromosome	3.88
R-HSA-72766	Reactome gene sets	Translation	3.79
GO:0033146	GO biological processes	Regulation of intracellular estrogen receptor signaling pathway	3.3
R-HSA-1428517	Reactome gene sets	The citric acid (TCA) cycle and respiratory electron transport	3.16
GO:0000209	GO biological processes	Protein polyubiquitination	2.75
GO:0034660	GO biological processes	ncRNA metabolic process	2.73
GO:0007059	GO biological processes	Chromosome segregation	2.67
GO:0051298	GO biological processes	Centrosome duplication	2.6
GO:0031123	GO biological processes	RNA 3′-end processing	2.52
GO:0006353	GO biological processes	DNA-templated transcription, termination	2.48
GO:0051604	GO biological processes	Protein maturation	2.3
R-HSA-8953854	Reactome gene sets	Metabolism of RNA	2.26
GO:1902882	GO biological processes	Regulation of response to oxidative stress	2.12

γH2A is an established marker of DNA damage (23). Relative to mock-silenced cells, DCTPP1-deficient induced BRCA cells had significantly higher γH2A levels (Figure 5B). These data indicate that DCTPP1-deficient accelerated DNA damage repair.

Regarding the functional analysis of these core modules (Figure 5A and Table 2), they are enriched in the DNA repair signaling pathway. DNA repair signaling influences cancer progression (24). Western blot analysis of the DNA repair signaling pathway factors, XRCC1, PARP1, and RAD51, revealed their elevation upon DCTPP1 upregulation in BRCA cells (Figure 5C).

**Table 2:** Functional terms enriched of modules in DCTPP1 co-expressing network.

**MCODE**	**GO**	**Description**	**–Log10(*P*)**
MCODE_1	R-HSA-5389840	Mitochondrial translation elongation	9.8
MCODE_1	GO:0070125	mitochondrial translational elongation	9.8
MCODE_1	R-HSA-5368287	Mitochondrial translation	9.7
MCODE_2	CORUM:351	Spliceosome	6.7
MCODE_2	R-HSA-72163	mRNA splicing—major pathway	6.4
MCODE_2	R-HSA-72172	mRNA splicing	6.3

### DCTPP1 Is a miR-378a-3p Direct Target in BRCA

**Figure 6:**
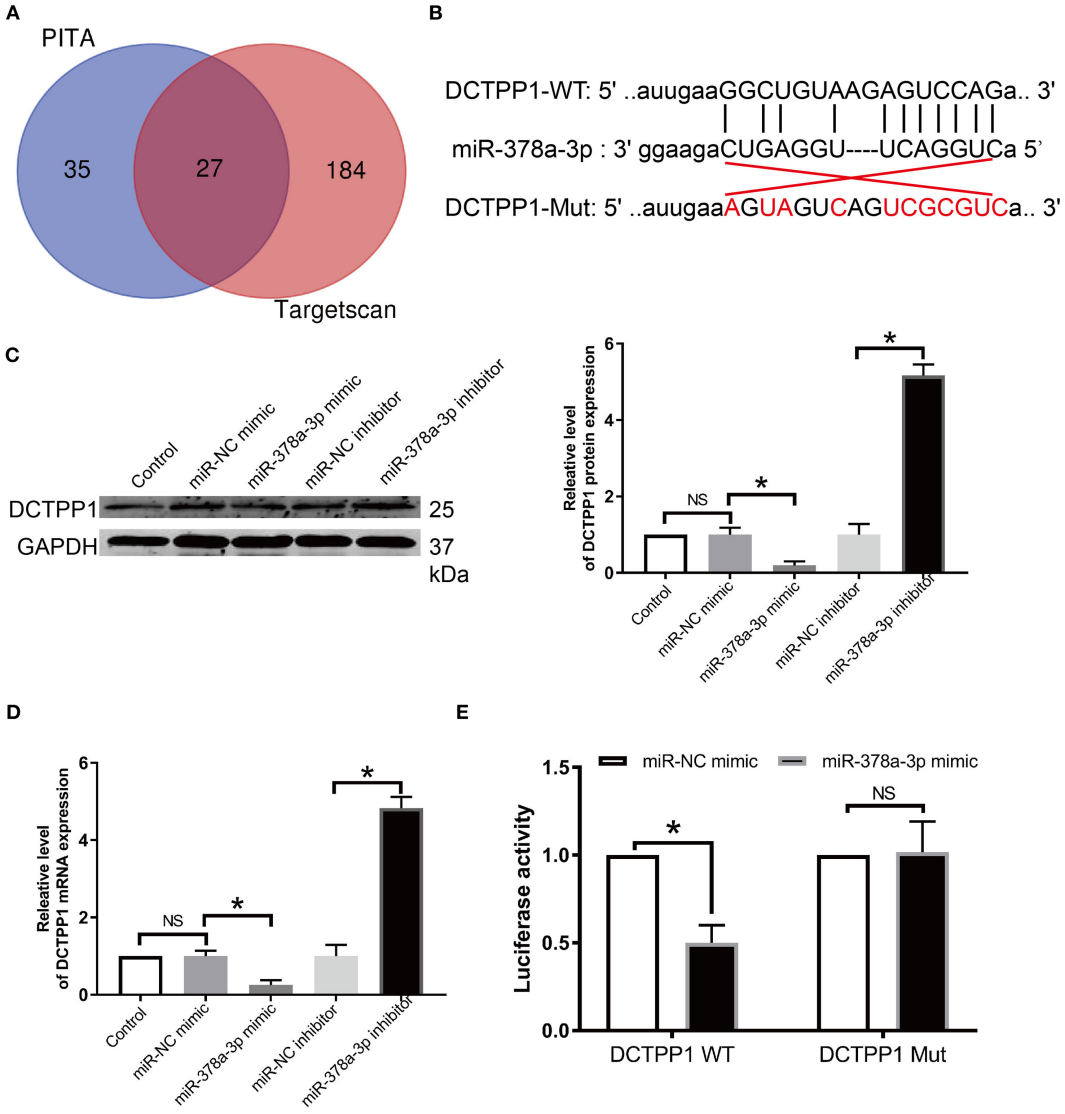
*DCTPP1* is directly regulated by miR-378a-3p. **(A)** MiR-378a-3p is potentially upstream of DCTPP1. **(B)** Seed unit between miR-378a-3p and DCTPP1. **(C)** DCTPP1 protein levels after transfection with miR-378a-3p mimic or suppressor. **(D)** RT-qPCR analysis of DCTPP1 expression upon transfection with miR-378a-3p mimic or suppressor. **(E)** Luciferase assay. All data are representative of at least three experiments. The results are presented as mean ± s.e.m. *p ≤ 0.05 was considered significant.

Given that miRNAs are implicated in BRCA progression (9, 25), we screened the publicly available databases, TargetScan and PITA, for miRNAs that may modulate DCTPP1 and found that miR-378a-3p has an optional seed unit for DCTPP1 (Figures 6A,B). Relative to negative controls, RT-qPCR and western blot analyses revealed DCTPP1 suppression in all the cell lines under miR-378a-3p overexpression (Figures 6C,D). Moreover, luciferase enzyme activity was repressed by miR-378a-3p in BRCA cells transfected with wild-type DCTPP1 3′-UTR (Figure 6E).

### miR-378a-3p Upregulation Inhibits BRCA Proliferation

**Figure 7:**
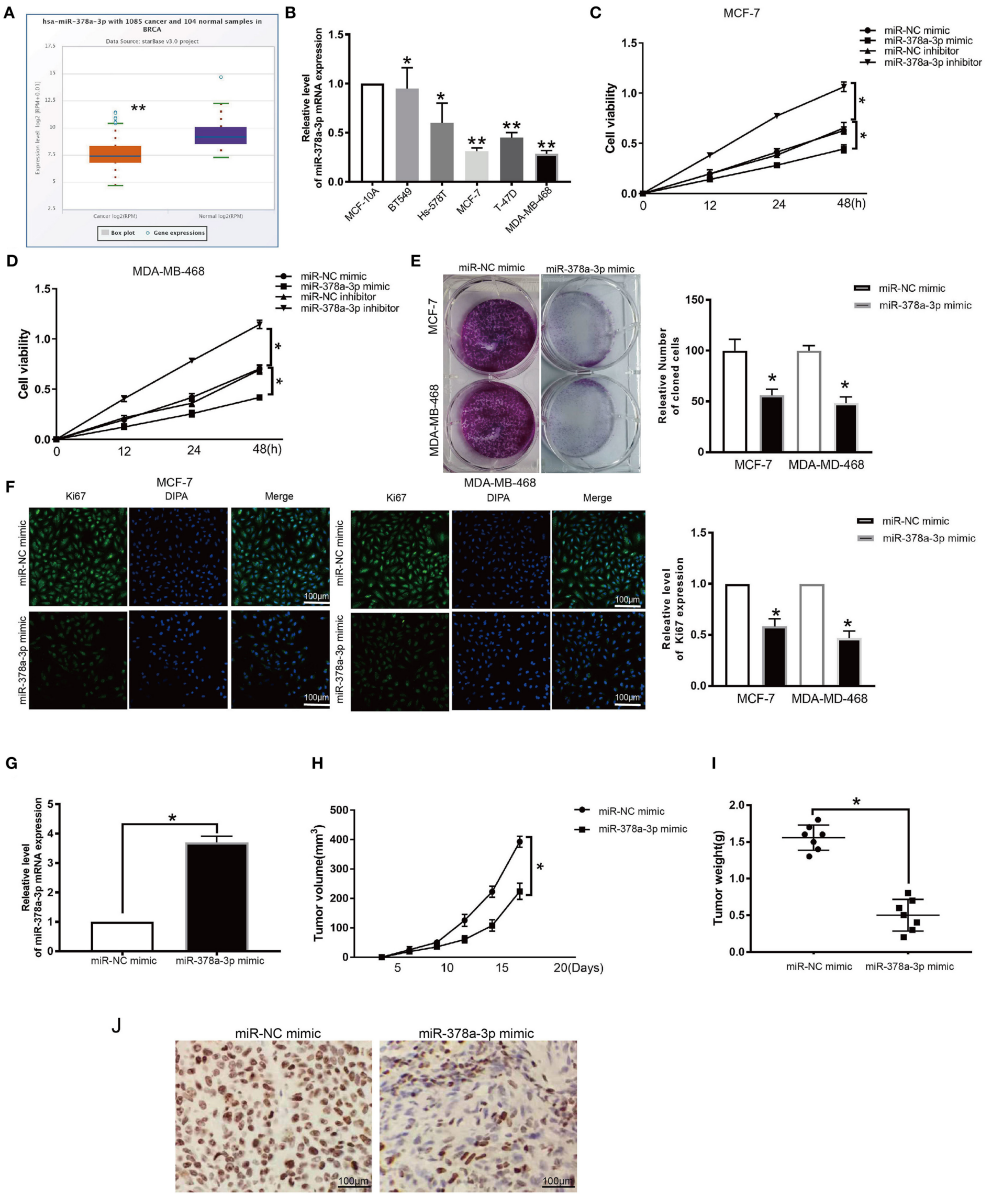
miR-378a-3p inhibits proliferation of BRCA cells. **(A)** MiR-378a-3p expression in BRCA patients TCGA datasets. **(B)** MiR-378a-3p expression in BRCA cells. **(C,D)** CCK-8 assay of BRCA cell growth upon transfection with miR-378a-3p mimic or inhibitor. **(E)** Colony formation assay of BRCA cells upon transfection with miR-378a-3p mimic or control. **(F)** Ki67 analysis of BRCA cell growth upon transfection with miR-378a-3p mimic or control. **(G)** MiR-378a-3p expression in tumors upon transfection with miR-378a-3p mimic. **(H)** tumor weight. **(I)** tumor volume. **(J)** Ki67 levels in tumors transfected with miR-378a-3p mimic. All data are representative of at least three experiments. Data are presented as mean ± SEM. *p ≤ 0.05, **p ≤ 0.01.

Evaluation of the expression of miR-378a-3p in BRCA patients' TCGA datasets revealed that it is significantly downregulated (*p* ≤ 0.05; Figure 7A). Moreover, we established that the expression of miR-378a-3p was dramatically lower in BRCA cell lines relative to MCF-10A (Figure 7B). To assess the function of miR-378a-3p in BRCA progression, we transfected the MCF-7 cells and MDA-MB-468 cells, which have high miR-378a-3p measures with the mimic of miR-378a-3p, and those with the lowest expression of miR-378a-3p with miR-378a-3p repressor (Supplementary Figure 2E). CCK-8 analysis revealed that the silencing of miR-378a-3p enhanced cell growth, while its overexpression markedly suppressed the growth of MCF-7 cells and MDA-MD-468 cells (Figures 7C,D). Consistent with these data, colony formation assay and ki67 staining indicated suppressed BRCA cell growth upon miR-378a-3p overexpression (Figures 7E,F). A mouse tumor xenograft revealed higher miR-378a-3p levels in miR-378a-3p mimic bearing tumors relative to the NC group (Figure 7G). The average number of harvested nodules (Figure 7H) and tumor weight (Figure 7I) were significantly lower in miR-378a-3p mimic bearing mice relative to the NC group. IHC analysis of Ki67 expression revealed lower ki67 levels in miR-378a-3p mimic bearing tumors relative to the NC group (Figure 7J). These data demonstrate that miR-378a-3p negatively modulates DCTPP1 in BRCA and that miR-378a-3p suppresses BRCA development *in vitro* and *in vivo*.

## Discussion

Although considerable progress has been made in the development of BRCA diagnostics, as well as prognostic biosignatures, our knowledge of the molecular mechanisms underlying BRCA is still lacking. The present study found that DCTPP1 is upregulated in BRCA and that its expression strongly correlates with BRCA progression. Our data demonstrate that DCTPP1 has an oncogenic role associated with DNA repair signaling in BRCA cells and that miR-378a-3p negatively modulates it.

Mounting evidence suggests that elevated DCTPP1 expression is tumorigenic (26, 27). Herein, we investigated the expression of DCTPP1 in multiple BRCA datasets on TCGA and GEO. Figure 1A shows the DCTPP1 expression profile in 33 cancers (GEPIA analysis). The UALCAN online tool analysis revealed that DCTPP1 transcription levels were remarkably different in BRCA patients with different ages, genders, disease stages, and nodal metastasis (Figure 2). Especially in different BC types, expression of DCTPP1 was higher in the Luminal type than that in other types (Figure 2G). Notably, *DCTPP1* expression significantly impacts BRCA prognosis [OS HR = 1.9, Logrank *p* = 0.00018, *p*(HR) = 0.00021; Figure 3A]. Our xenograft mouse model of BRCA showed that DCTPP1 silencing suppresses tumor growth and *in vitro* assays confirmed that DCTPP1 silencing suppresses BRCA cell proliferation.

DCTPP1 has been reported to play an important role in DNA damage and genetic instability in both chromosomal and mitochondrial DNA in apoptosis and DNA repair (5, 27). Our data showed that DCTPP1 silencing triggers DNA damage in BRCA cells, while its upregulation up-regulated the levels of DNA repair-associated factors, XRCC1, PARP1, and RAD51. DNA repair pathways are DNA damage response mechanisms (28, 29). DNA repair pathways influence cell survival and are anti-cancer therapeutic targets of radiotherapy and cytotoxic chemotherapy (30, 31). γh2A is a major agent in DNA damage or repair (32). Our data showed that silencing DCTPP1 up-regulates γ-H2AX expression. XRCC1 is reported to be activated via DNA damage, initiating cellular signaling cascades of great importance (33, 34). Multiple studies suggest that hepatocarcinogenesis is triggered by aggregated lesions, including chromosomal aberrations and DNA damage as a result of impaired DNA damage response and dysregulated DNA damage repair (35). Further studies are needed to determine if miRNAs regulate DCTPP1, which may allude to a modulatory feedback mechanism between these two factors.

miRNAs regulate the expression of their target genes in the post-transcriptional stage (36, 37) and play crucial roles in oncogenesis and loss of tumor suppression, they are implicated in multiple human cancers (38, 39). Herein, we demonstrated that miR-378a-3p serves as an upstream modulator of DCTPP1 and, using luciferase studies, found that it directly targets DCTPP1. MiR-378a-3p is dysregulated in some cancers, where it has tumor-suppressor functions (40–42). We find that miR-378a-3p is downregulated in BRCA tissue, and its expression is negatively linked to tumor size. Moreover, we find it suppresses BRCA proliferation via DCTPP1/DNA repair signaling.

In summary, our data identify DCTPP1 as an oncogene in BRCA. miR-378a-3p as an upstream gene of DCTPP1 demonstrated the way that DCTPP1 modulates targeted genes and DNA repair cascades. Our findings that DCTPP1 controls BRCA proliferation uncover novel potential therapeutic strategies against BRCA.

## Data Availability Statement

The raw data supporting the conclusions of this article will be made available by the authors, without undue reservation.

## Ethics Statement

All animal experiments adhered to guidelines by Institutional Animal Care and Use Committee of the Harbin Medical University.

## Author Contributions

MN, MS, YL, and YS: conception and design. YL, YS, and J-gH: administrative support. MN, MS, and SS: provision of study materials or patients. MN, MS, and G-qZ: collection and assembly of data. MN, MS, YL, YS, J-gH, SS, X-sL, and G-qZ: data analysis and interpretation. All authors: manuscript writing and final approval of manuscript.

## Conflict of Interest

The authors declare that the research was conducted in the absence of any commercial or financial relationships that could be construed as a potential conflict of interest. The handling editor declared a shared affiliation with the authors at time of review.

## Supplementary Material

Supplementary Table 1 Primer sequences.

Supplementary Figure 1 Expression of DCTPP1 in different BC cells. **(A,B)** DCTPP1 expression in different BC cell lines. Results are presented as mean ± s.e.m. ^*^*p* ≤ 0.05 in contrast with the controls (MCF-10A).

Supplementary Figure 2 *DCTPP1* and miR-378a-3p expression in BRCA cells. **(A,B)** DCTPP1 expression upon DCTPP1 silencing. **(C,D)** DCTPP1 expression upon DCTPP1 over-expression. **(E)** miR-378a-3p levels upon transfection with miR-378a-3p mimic, inhibitor, or NC. Results are presented as mean ± s.e.m. ^**^*p* ≤ 0.01, ^*^*p* ≤ 0.05 in contrast with the controls, ^NS^*p* ≥ 0.05 vs. controls.

## References

[1] Li J Yu K Pang D Wang C Jiang J Yang S. Adjuvant capecitabine with docetaxel and cyclophosphamide plus epirubicin for triple-negative breast cancer (CBCSG010): an open-label, randomized, multicenter, phase III trial. J Clin Oncol. (2020) 38:1774–84. 10.1200/JCO.19.02474 32275467 PMC7255982

[2] Xu S Liu H Wan L Zhang W Wang Q Zhang S. The MS-lincRNA landscape reveals a novel lincRNA BCLIN25 that contributes to tumorigenesis by upregulating ERBB2 expression via epigenetic modification and RNA-RNA interactions in breast cancer. Cell Death Dis. (2019) 10:920. 10.1038/s41419-019-2137-5 31801944 PMC6892920

[3] Shao Z Pang D Yang H Li W Wang S Cui S. Efficacy, safety, and tolerability of pertuzumab, trastuzumab, and docetaxel for patients with early or locally advanced ERBB2-positive breast cancer in Asia: the PEONY phase 3 randomized clinical trial. JAMA Oncol. (2019) 6:e193692. 10.1001/jamaoncol.2019.3692 31647503 PMC6813591

[4] Zhang J Sui S Wu H Zhang J Zhang X Xu S. The transcriptional landscape of lncRNAs reveals the oncogenic function of LINC00511 in ER-negative breast cancer. Cell Death Dis. (2019) 10:599. 10.1038/s41419-019-1835-3 31395854 PMC6687715

[5] Martinez-Arribas B Requena CE Perez-Moreno G Ruiz-Perez LM Vidal AE Gonzalez-Pacanowska D. DCTPP1 prevents a mutator phenotype through the modulation of dCTP, dTTP and dUTP pools. Cell Mol Life Sci. (2020) 77:1645–60. 10.1007/s00018-019-03250-x 31377845 PMC7162842

[6] Song FF Xia LL Ji P Tang YB Huang ZM Zhu L. Human dCTP pyrophosphatase 1 promotes breast cancer cell growth and stemness through the modulation on 5-methyl-dCTP metabolism and global hypomethylation. Oncogenesis. (2015) 4:e159. 10.1038/oncsis.2015.10 26075750 PMC4491611

[7] Zauri M Berridge G Thezenas ML Pugh KM Goldin R Kessler BM. CDA directs metabolism of epigenetic nucleosides revealing a therapeutic window in cancer. Nature. (2015) 524:114–8. 10.1038/nature14948 26200337 PMC4866471

[8] Zhang Y Ye WY Wang JQ Wang SJ Ji P Zhou GY. dCTP pyrophosphohydrase exhibits nucleic accumulation in multiple carcinomas. Eur J Histochem. (2013) 57:e29. 10.4081/ejh.2013.e29 24085278 PMC3794360

[9] Nassar FJ Nasr R Talhouk R. MicroRNAs as biomarkers for early breast cancer diagnosis, prognosis and therapy prediction. Pharmacol Ther. (2017) 172:34–49. 10.1016/j.pharmthera.2016.11.012 27916656

[10] Fridrichova I Zmetakova I. MicroRNAs contribute to breast cancer invasiveness. Cells. (2019) 8:1361. 10.3390/cells8111361 31683635 PMC6912645

[11] Bertoli G Cava C Castiglioni I. MicroRNAs: new biomarkers for diagnosis, prognosis, therapy prediction and therapeutic tools for breast cancer. Theranostics. (2015) 5:1122–43. 10.7150/thno.11543 26199650 PMC4508501

[12] Liu Q Peng F Chen J. The role of exosomal microRNAs in the tumor microenvironment of breast cancer. Int J Mol Sci. (2019) 20:3884. 10.3390/ijms20163884 31395836 PMC6719057

[13] Wang B Mao JH Wang BY Wang LX Wen HY Xu LJ. Exosomal miR-1910-3p promotes proliferation, metastasis, and autophagy of breast cancer cells by targeting MTMR3 and activating the NF-kappaB signaling pathway. Cancer Lett. (2020) 489:87–99. 10.1016/j.canlet.2020.05.038 32531321

[14] Elango R Alsaleh KA Vishnubalaji R Manikandan M Ali AM Abd El-Aziz N. MicroRNA expression profiling on paired primary and lymph node metastatic breast cancer revealed distinct microRNA profile associated With LNM. Front Oncol. (2020) 10:756. 10.3389/fonc.2020.00756 32509578 PMC7248321

[15] Rinnerthaler G Gampenrieder SP Hackl H Steiner M Monzo-Fuentes C Melchardt T. Low expression of miR-20a-5p predicts benefit to bevacizumab in metastatic breast cancer patients treated within the TANIA phase III trial. J Clin Med. (2020) 9:1663. 10.3390/jcm9061663 32492882 PMC7355487

[16] Zhu L Jiang S Yu S Liu X Pu S Xie P. Increased SIX-1 expression promotes breast cancer metastasis by regulating lncATB-miR-200s-ZEB1 axis. J Cell Mol Med. (2020) 24:5290–303. 10.1111/jcmm.15185 32227618 PMC7205823

[17] Guo XB Zhang XC Chen P Ma LM Shen ZQ. miR378a3p inhibits cellular proliferation and migration in glioblastoma multiforme by targeting tetraspanin 17. Oncol Rep. (2019) 42:1957–71. 10.3892/or.2019.7283 31432186 PMC6775804

[18] Xu ZH Yao TZ Liu W. miR-378a-3p sensitizes ovarian cancer cells to cisplatin through targeting MAPK1/GRB2. Biomed Pharmacother. (2018) 107:1410–7. 10.1016/j.biopha.2018.08.132 30257357

[19] Dalmasso B Hatse S Brouwers B Laenen A Berben L Kenis C. Age-related microRNAs in older breast cancer patients: biomarker potential and evolution during adjuvant chemotherapy. BMC Cancer. (2018) 18:1014. 10.1186/s12885-018-4920-6 30348127 PMC6196565

[20] Chatterjee N Walker GC. Mechanisms of DNA damage, repair, and mutagenesis. Environ Mol Mutagen. (2017) 58:235–63. 10.1002/em.22087 28485537 PMC5474181

[21] Li C Han J Yao Q Zou C Xu Y Zhang C. Subpathway-GM: identification of metabolic subpathways via joint power of interesting genes and metabolites and their topologies within pathways. Nucleic Acids Res. (2013) 41:e101. 10.1093/nar/gkt161 23482392 PMC3643575

[22] Qiao K Ning S Wan L Wu H Wang Q Zhang X. LINC00673 is activated by YY1 and promotes the proliferation of breast cancer cells via the miR-515-5p/MARK4/Hippo signaling pathway. J Exp Clin Cancer Res. (2019) 38:418. 10.1186/s13046-019-1421-7 31623640 PMC6796384

[23] Chen F Xu B Li J Yang X Gu J Yao X. Hypoxic tumour cell-derived exosomal miR-340-5p promotes radioresistance of oesophageal squamous cell carcinoma via KLF10. J Exp Clin Cancer Res. (2021) 40:38. 10.1186/s13046-021-01834-9 33485367 PMC7825246

[24] Sobczak M Pitt AR Spickett CM Robaszkiewicz A. PARP1 co-regulates EP300-BRG1-dependent transcription of genes involved in breast cancer cell proliferation and DNA repair. Cancers (Basel). (2019) 11:1539. 10.3390/cancers11101539 31614656 PMC6826995

[25] Rahman MM Brane AC Tollefsbol TO. MicroRNAs and epigenetics strategies to reverse breast cancer. Cells. (2019) 8:1214. 10.3390/cells8101214 31597272 PMC6829616

[26] Jordaens S Cooksey L Bonney S Orchard L Coutinho M Van Tendeloo V. Serum profiling identifies ibrutinib as a treatment option for young adults with B-cell acute lymphoblastic leukaemia. Br J Haematol. (2020) 189:500–12. 10.1111/bjh.16407 32064588

[27] Scaletti E Claesson M Helleday T Jemth AS Stenmark P. The first structure of an active mammalian dCTPase and its complexes with substrate analogs and products. J Mol Biol. (2020) 432:1126–42. 10.1016/j.jmb.2020.01.005 31954130

[28] Xie C Li N Wang H He C Hu Y Peng C. Inhibition of autophagy aggravates DNA damage response and gastric tumorigenesis via Rad51 ubiquitination in response to *H. pylori* infection. Gut Microbes. (2020) 11:1567–89. 10.1080/19490976.2020.1774311 32588736 PMC7524160

[29] Suo D Wang Z Li L Chen Q Zeng T Liu R. HOXC10 upregulation confers resistance to chemoradiotherapy in ESCC tumor cells and predicts poor prognosis. Oncogene. (2020) 39:5441–54. 10.1038/s41388-020-1375-4 32587398

[30] Wang D Zhou Z Wu E Ouyang C Wei G Wang Y. LRIK interacts with the Ku70-Ku80 heterodimer enhancing the efficiency of NHEJ repair. Cell Death Differ. (2020) 27:3337–53. 10.1038/s41418-020-0581-5 32587379 PMC7852670

[31] Carraway HE Malkaram SA Cen Y Shatnawi A Fan J Ali HEA. Activation of SIRT6 by DNA hypomethylating agents and clinical consequences on combination therapy in leukemia. Sci Rep. (2020) 10:10325. 10.1038/s41598-020-67170-8 32587297 PMC7316973

[32] Wang T Tu Y Wang K Gong S Zhang G Zhang Y. Associations of blood lead levels with multiple genotoxic biomarkers among workers in China: a population-based study. Environ Pollut. (2020) 273:116181. 10.1016/j.envpol.2020.116181 33508628

[33] Rajagopal T Seshachalam A Rathnam KK Jothi A Viswanathan S Talluri S. DNA repair genes hOGG1, XRCC1 and ERCC2 polymorphisms and their molecular mapping in breast cancer patients from India. Mol Biol Rep. (2020) 47:5081–90. 10.1007/s11033-020-05577-2 32519309

[34] Kalasova I Hailstone R Bublitz J Bogantes J Hofmann W Leal A. Pathological mutations in PNKP trigger defects in DNA single-strand break repair but not DNA double-strand break repair. Nucleic Acids Res. (2020) 48:6672–84. 10.1093/nar/gkaa489 32504494 PMC7337934

[35] Kaiser RWJ Erber J Hopker K Fabretti F Muller RU. AATF/Che-1-An RNA binding protein at the nexus of DNA damage response and ribosome biogenesis. Front Oncol. (2020) 10:919. 10.3389/fonc.2020.00919 32587828 PMC7298124

[36] Okuno J Miyake T Sota Y Tanei T Kagara N Naoi Y. Development of prediction model including microRNA expression for sentinel lymph node metastasis in ER-positive and HER2-negative breast cancer. Ann Surg Oncol. (2020) 28:310–9. 10.1245/s10434-020-08735-9 32583195

[37] Gibadulinova A Bullova P Strnad H Pohlodek K Jurkovicova D Takacova M. CAIX-mediated control of LIN28/let-7 axis contributes to metabolic adaptation of breast cancer cells to hypoxia. Int J Mol Sci. (2020) 21:4299. 10.3390/ijms21124299 32560271 PMC7352761

[38] Chen Y Wu N Liu L Dong H Liu X. microRNA-128-3p overexpression inhibits breast cancer stem cell characteristics through suppression of Wnt signalling pathway by down-regulating NEK2. J Cell Mol Med. (2020) 24:7353–69. 10.1111/jcmm.15317 32558224 PMC7339185

[39] Zhang Q Li T Wang Z Kuang X Shao N Lin Y. lncRNA NR2F1-AS1 promotes breast cancer angiogenesis through activating IGF-1/IGF-1R/ERK pathway. J Cell Mol Med. (2020)24:8236–47. 10.1111/jcmm.15499 32548873 PMC7348140

[40] Huang X Yang Y Yang C Li H Cheng H Zheng Y. Overexpression of LBX2 associated with tumor progression and poor prognosis in colorectal cancer. Oncol Lett. (2020) 19:3751–60. 10.3892/ol.2020.11489 32382328 PMC7202318

[41] Zhang Y Yu R Li L. LINC00641 hinders the progression of cervical cancer by targeting miR-378a-3p/CPEB3. J Gene Med. (2020) 22:e3212. 10.1002/jgm.3212 32367630

[42] Briand J Garnier D Nadaradjane A Clement-Colmou K Potiron V Supiot S. Radiotherapy-induced overexpression of exosomal miRNA-378a-3p in cancer cells limits natural killer cells cytotoxicity. Epigenomics. (2020) 12:397–408. 10.2217/epi-2019-0193 32267172

