# Supplementary material for: DCTPP1, an Oncogene Regulated by miR-378a-3p, Promotes Proliferation of Breast Cancer via DNA Repair Signaling Pathway
Source: Front Oncol. 2021 May 25;11:641931. doi: 10.3389/fonc.2021.641931 (PMC8185175; doi:10.3389/fonc.2021.641931)
Supplement: Supplementary Table 1 — Primer sequences. [file Table_1.DOCX]

**Table S1 Primer sequence**

| DCTPP1: | Forward, 5ʹ- CGCCTCCATGCTGAGTTTG-3ʹ, | Reverse, 5ʹ- CCAGGTTCCCCATCGGTTTTC-3ʹ; |
| --- | --- | --- |
| miR-378-3p: | Forward, 5ʹ - AAGGTGAAGGTCGGAGTCAAC -3ʹ, | Reverse, 5ʹ- GCTGTCAACGATACGCTACGTAACG -3ʹ; |
| **GAPDH:** | Forward, 5’- GAAGAGCTACGAGCTGCCTGA-3ʹ, | Reverse, 5ʹ- GGGGTCATTGATGGCAACAATA-3ʹ |
| **U6:** | Forward, 5ʹ- CTCGCTTCGGCAGCACA -3ʹ, | Reverse, 5ʹ- AACGCTTCACGAATTTGCGT -3ʹ |
