# Supplementary figures and images for: DCTPP1, an Oncogene Regulated by miR-378a-3p, Promotes Proliferation of Breast Cancer via DNA Repair Signaling Pathway
Source: Front Oncol. 2021 May 25;11:641931. doi: 10.3389/fonc.2021.641931 (PMC8185175; doi:10.3389/fonc.2021.641931)

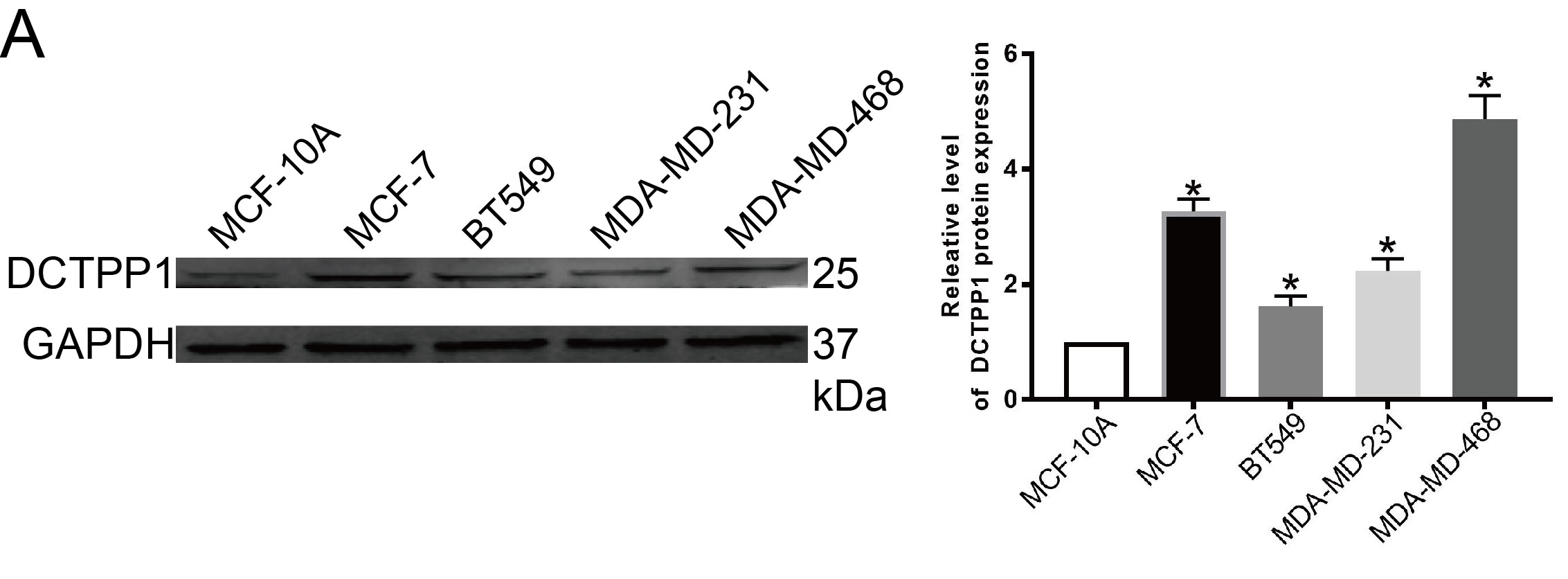

Supplement: Supplementary Figure 1 — Expression of DCTPP1 in different BC cells. (A,B) DCTPP1 expression in different BC cell lines. Results are presented as mean ± s.e.m. *p ≤ 0.05 in contrast with the controls (MCF-10A). [file Image_1.JPEG]

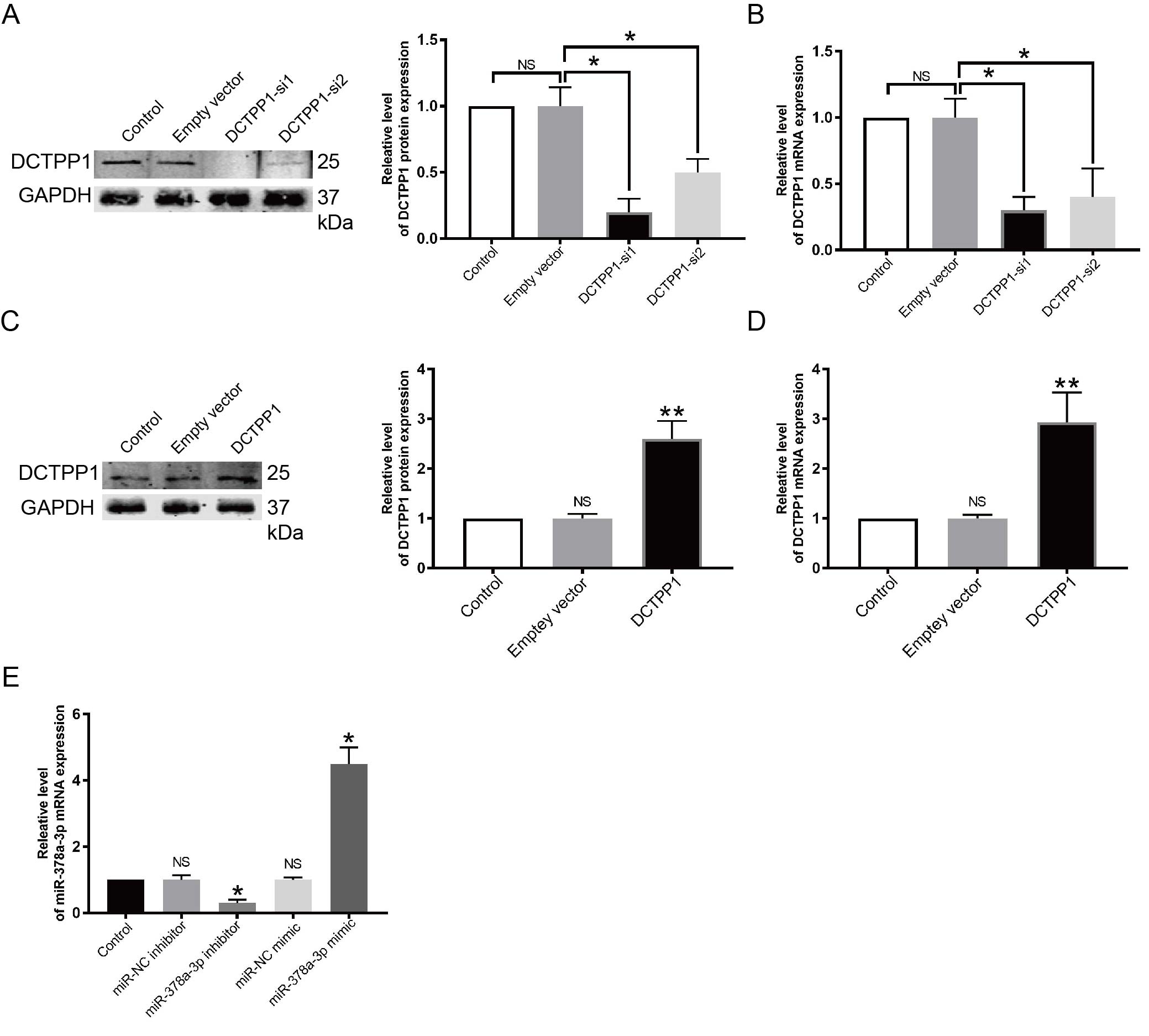

Supplement: Supplementary Figure 2 — DCTPP1 and miR-378a-3p expression in BRCA cells. (A,B) DCTPP1 expression upon DCTPP1 silencing. (C,D) DCTPP1 expression upon DCTPP1 over-expression. (E) miR-378a-3p levels upon transfection with miR-378a-3p mimic, inhibitor, or NC. Results are presented as mean ± s.e.m. **p ≤ 0.01, *p ≤ 0.05 in contrast with the controls, NSp ≥ 0.05 vs. controls. [file Image_2.JPEG]
